# A Case of Human Intestinal Spirochetosis Diagnosed During Screening Colonoscopy

**DOI:** 10.7759/cureus.14829

**Published:** 2021-05-04

**Authors:** Lynna Alnimer, Ali Zakaria, Bradley Warren

**Affiliations:** 1 Department of Internal Medicine, Ascension Providence Hospital, Michigan State University/College of Human Medicine, Southfield, USA; 2 Department of Gastroenterology, Ascension Providence Hospital, Michigan State University/College of Human Medicine, Southfield, USA

**Keywords:** colonic biopsy, human intestinal spirochetosis, spirochetes, spirochetosis, colonoscopy

## Abstract

Human intestinal spirochetosis (HIS) is a rare disease and mostly encountered incidentally during colorectal cancer screening colonoscopy. Risk factors include homosexuality and immunocompromised states. Patients are usually asymptomatic; however, chronic diarrhea and bloody stools have been reported in some cases. Diagnosis is usually confirmed by histopathology. A watch-and-see approach is usually acceptable, but successful treatment with Metronidazole has been reported in symptomatic cases. Its clinical significance remains questionable given that patients are mostly asymptomatic.

## Introduction

Human intestinal spirochetosis (HIS) was first described by Harland and Lee [1]. It is also known as colonic spirochetosis and refers to the colonization of the colorectal epithelium by anaerobic spirochetes, namely *Brachyspira aalborgi *and *Brachyspira pilosicoli *[2]. HIS is usually identified by pathologists in colonic biopsies but its clinical significance remains controversial [3]. Most patients are asymptomatic; however, some patients, especially immunocompromised, may present with chronic diarrhea, constipation, hematochezia, and abdominal pain [4]. We report a case of HIS in an asymptomatic 45-year-old male diagnosed incidentally during average risk colorectal cancer screening colonoscopy.

## Case presentation

**Figure 1:**
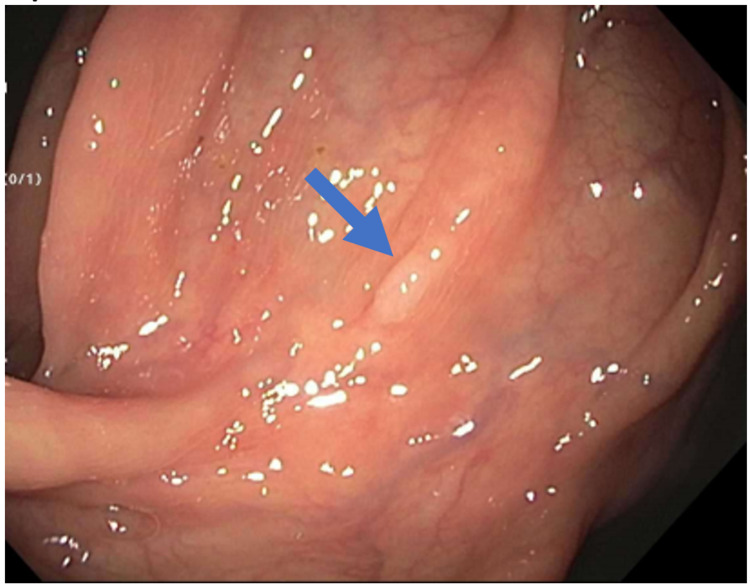
Colonoscopy view showing sessile polyp (marked with blue arrow).

This was a 45-year-old Hispanic homosexual male who presented to our endoscopy unit for an average risk colorectal cancer screening evaluation. He denied having any symptoms. Colonoscopy revealed a total of four sessile diminutive polyps; two in the descending colon, one in the transverse colon, and one in the ascending colon (Figure 1), all resected and retrieved with cold biopsy forceps.

**Figure 2:**
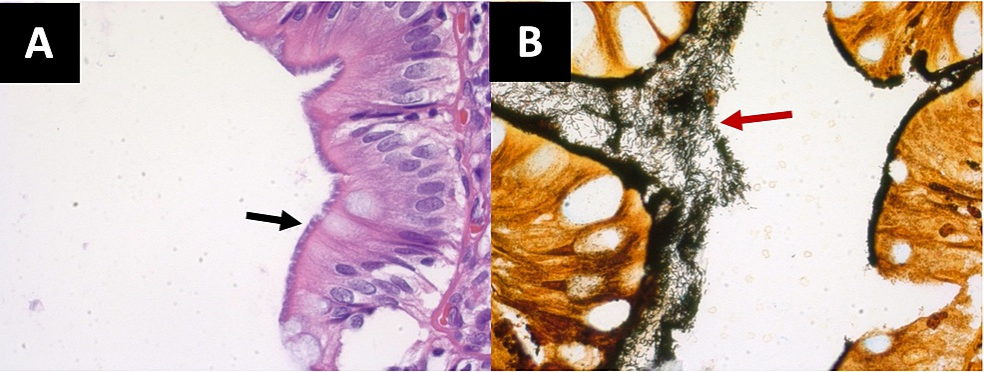
Histopathological findings with hematoxylin-eosin and Warthin-Starry staining. (A) Hematoxylin-eosin stain (60×) highlighting filamentous structures on the epithelial surface of the colonic polyp biopsy (black arrow). (B) Spirochetes (red arrow) appear black with Warthin-Starry (Silver) stain (60×), consistent with human intestinal spirochetosis.

Pathology showed hyperplastic polyps and Warthin Starry stain was positive for intestinal spirochetosis (Figure 2).

Given the histopathological findings, the patient was referred to an infectious disease specialist. Rapid plasma regains (RPR) and human immunodeficiency virus (HIV) were negative. The decision was to monitor the clinical condition and avoid antibiotics for the time being, given the absence of symptoms.

## Discussion

Intestinal spirochetosis is common in the veterinary world causing significant symptoms yet remains a controversial topic in human medicine [5]. The prevalence of HIS in rectal biopsy specimens in Western countries is between 2% and 7% [6,7]. On the other hand, the prevalence is higher in patients originating from Asia, Southern India, and West Africa [6]. In one retrospective study by Weisheit et al., the average age at diagnosis reported was 51 years [8]. 

The highest prevalence rate is in homosexual men reaching up to 54% and in HIV-infected patients [9,10]. In the United States, homosexual males have shown rates of colonization up to 62.5% [10]. This prompted clinicians to consider whether HIS is sexually transmitted. Interestingly, there did not appear to be a correlation between the degree of immunodeficiency and the severity of symptoms [11].

HIS is usually an incidental finding in most cases. However, various symptoms have been reported with the most common being chronic (watery) diarrhea [4,8]. Constipation, alternating diarrhea and constipation, abdominal pain, and hematochezia are other symptoms that have been described. The mechanism of diarrhea is not well understood and is believed to be non-invasive secondary to destruction of microvilli thus loss of absorptive surface [12]. Very rarely, cases of spirochetemia have been reported in immunocompromised and critically ill patients [13].

Colonic involvement has been described from the proximal to the distal colon and in the vermiform appendix [14]. A single-institution study in Japan described preference to the right colon [15]. Alsaigh and Fogt examined 15 biopsies with histologically confirmed intestinal spirochetes and demonstrated various endoscopic appearances of colonic spirochetosis including a polypoid appearance in seven patients, a lesion in one patient, and an erythematous area in one patient [16]. Some patients had HIS in normal-appearing mucosa. Graham et al. reviewed biopsies from 33 patients with HIS, and 13 of them had colonic polyps [17]. Our patient had hyperplastic polyps that are similar to findings demonstrated in other studies.

Diagnosis is traditionally based on histological examination of colonic biopsy specimens by visualization of the microorganisms using the hematoxylin-eosin (H&E) stain under light microscopy. The abnormality is seen on the surface of the epithelium showing thread-like structures “blue fringe” in a palisade-like arrangement [5]. In addition, Warthin-Starry and Dieterle silver impregnation are other stains that can be used for further clarification [5]. On electron microscopy, the spirochetes tend to embed themselves in the colonic cells without invasion [1]. Spirochetes appear docking into the cell membrane in between destroyed microvilli [1]. Newer methods include polymerase chain reaction (PCR) amplification of a 16s rRNA to detect *B. pilosicoli* from human feces or colonic biopsy specimens and fluorescent in-situ hybridization (FISH) with oligonucleotide probes targeting 16S and 23S rRNA of *B. aalborgi* and *B. pilosicoli* [18]. 

In most cases, the patient is monitored without prescribing any medication. When the disease is causing severe symptoms, Metronidazole 500 mg four times a day for 10 days can be used to treat patients [19]. Moreover, some patients did not show improvement in symptoms despite the use of antibiotics which questions the clinical significance of the disease [5]. There has been symptomatic improvement in some cases with the use of other antibiotics such as clindamycin and macrolides [20].

## Conclusions

Human intestinal spirochetosis is a rare incidentally diagnosed infection yet its clinical significance remains indeterminate. HIS should be included in the differential diagnosis of immunocompromised patients presenting with chronic diarrhea. The surveillance interval colonic polyps with HIS remains an unanswered question. Since HIS is being more recognized with novel diagnostic techniques, it is anticipated that data will expand clarifying its clinical significance.
